# Supplementary material for: Donor regulatory T cells rapidly adapt to recipient tissues to control murine acute graft-versus-host disease
Source: Nat Commun. 2024 Apr 15;15:3224. doi: 10.1038/s41467-024-47575-z (PMC11018811; doi:10.1038/s41467-024-47575-z)
Supplement: Supplementary file 1 — Supplementary Information [file 41467_2024_47575_MOESM1_ESM.pdf]

Supplemental Information for

Donor regulatory T cells rapidly adapt to recipient tissues to control murine acute graft-versus-host disease

David J. Dittmar[1,3#], Franziska Pielmeier[1#], Nicholas Strieder[2], Alexander Fischer[1], Michael Herbst[1,4], Hanna Stanewsky[1], Niklas Wenzl[2], Eveline Röseler[2], Rüdiger Eder[1], Claudia Gebhard[2], Lucia Schwarzfischer-Pfeilschifter[1], Christin Albrecht[1], Wolfgang Herr[1], Matthias Edinger[1,2,§,*], Petra Hoffmann[1,2,§,*], Michael Rehli[1,2,§,*]

[1]Department of Internal Medicine III, University Hospital Regensburg, 93053 Regensburg, Germany
[2]Leibniz Institute for Immunotherapy, 93053 Regensburg, Germany
[3]present address: BioNTech SE, 82061 Neuried, Germany
[4]present address: Institute of Experimental Immunology, Research Unit Tumorimmunology, University Zurich, Switzerland

[#] These authors contributed equally
[§] These authors jointly supervised this work
[*]Corresponding authors: petra.hoffmann@ukr.de, matthias.edinger@ukr.de, michael.rehli@ukr.de

Supplement Index:

Supplemental Figures 1-7 page 02-13
Supplemental Tables 1-11 page 14-24
Supplemental References page 25

Supplemental Figures & Legends

Supplemental Figure 1

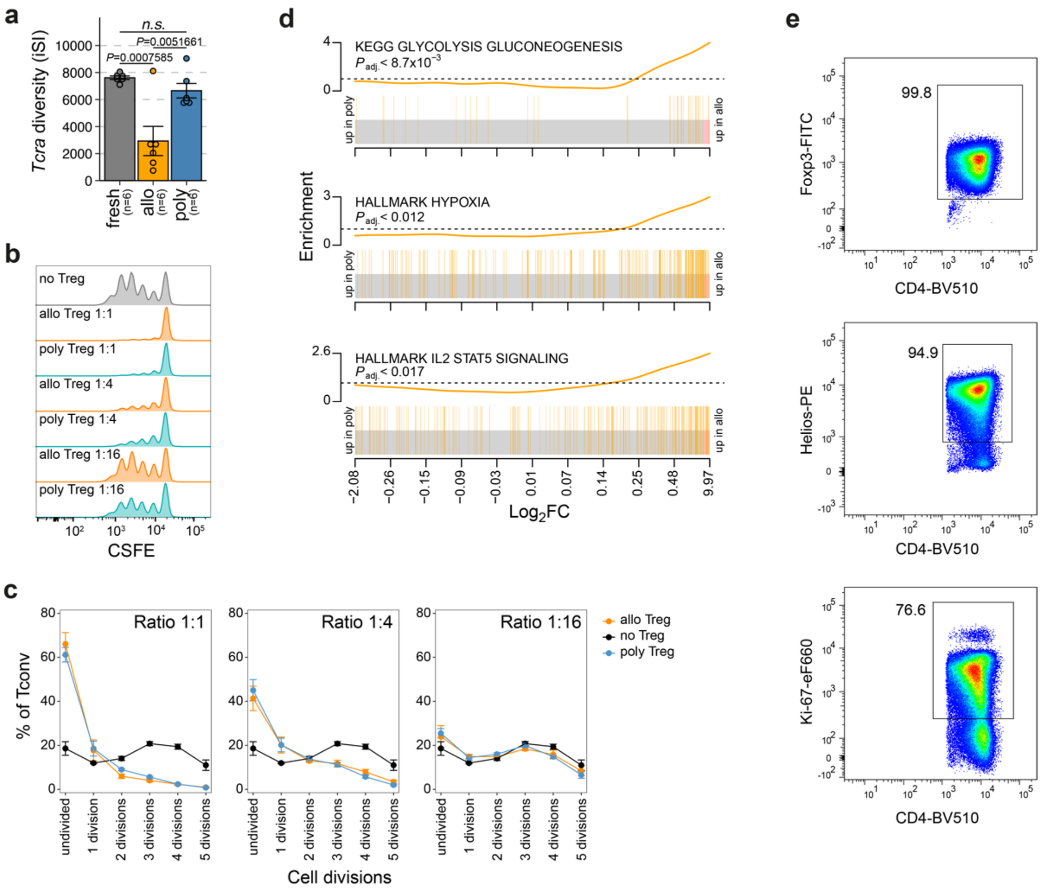

Polyclonal versus allogeneic expansion: properties of Treg products, Related to Figure 1. (a) Barplot of *TRA* clonotype diversities (iSI, inverse Simpson index) in independent isolates of splenic C57BL/6 Treg (fresh, d0) or in vitro expanded Treg products (allo, poly, d11-14) as measured by UMI-based sequencing of 5'RACE products of *TRA* mRNAs. Bars represent means±SEM of n=6 independent experiments, significant differences between groups are indicated above bars (1-way ANOVA with Tukey's post hoc test). Individual data points are shown as dots. (b) and (c) Treg suppression assay. *In vitro* expanded Treg (allo or poly) and CFSE-labelled congenic Tconv cells were co-cultured at the indicated ratios on autologous APC and stimulated with anti-CD3 for 3 days. Afterwards cells were harvested, stained for CD4 and congenic markers and analyzed by FACS using the 'proliferation' tool of FlowJo®. (b) Histograms show data from one representative experiment. (c) Combined data from n=4 independent Treg cultures per group. Data are presented as mean values±SEM (n=4). (d) Barcode plots presenting GSEA results (Hallmark and Kegg pathway associated genes) across the logFC ranking of genes based on the comparison of allo and poly Treg. Benjamini-Hochberg adjusted enrichment *P* values of competitive gene set tests are given. (e) Gating for Foxp3, Helios and Ki-67 in *in vitro* expanded Treg. Plots show cells from Treg cultures after gating for live single $CD4^+$ lymphocytes. Gates are based on marker expression in *ex vivo* live single $CD4^+$ T cells (Foxp3) or live single $CD4^+Foxp3^+$ T cells (Helios and Ki-67) isolated from spleens of $Foxp3^{gfp}$ donor mice. (a,c) Source data are provided as a Source Data file.

Supplemental Figure 2

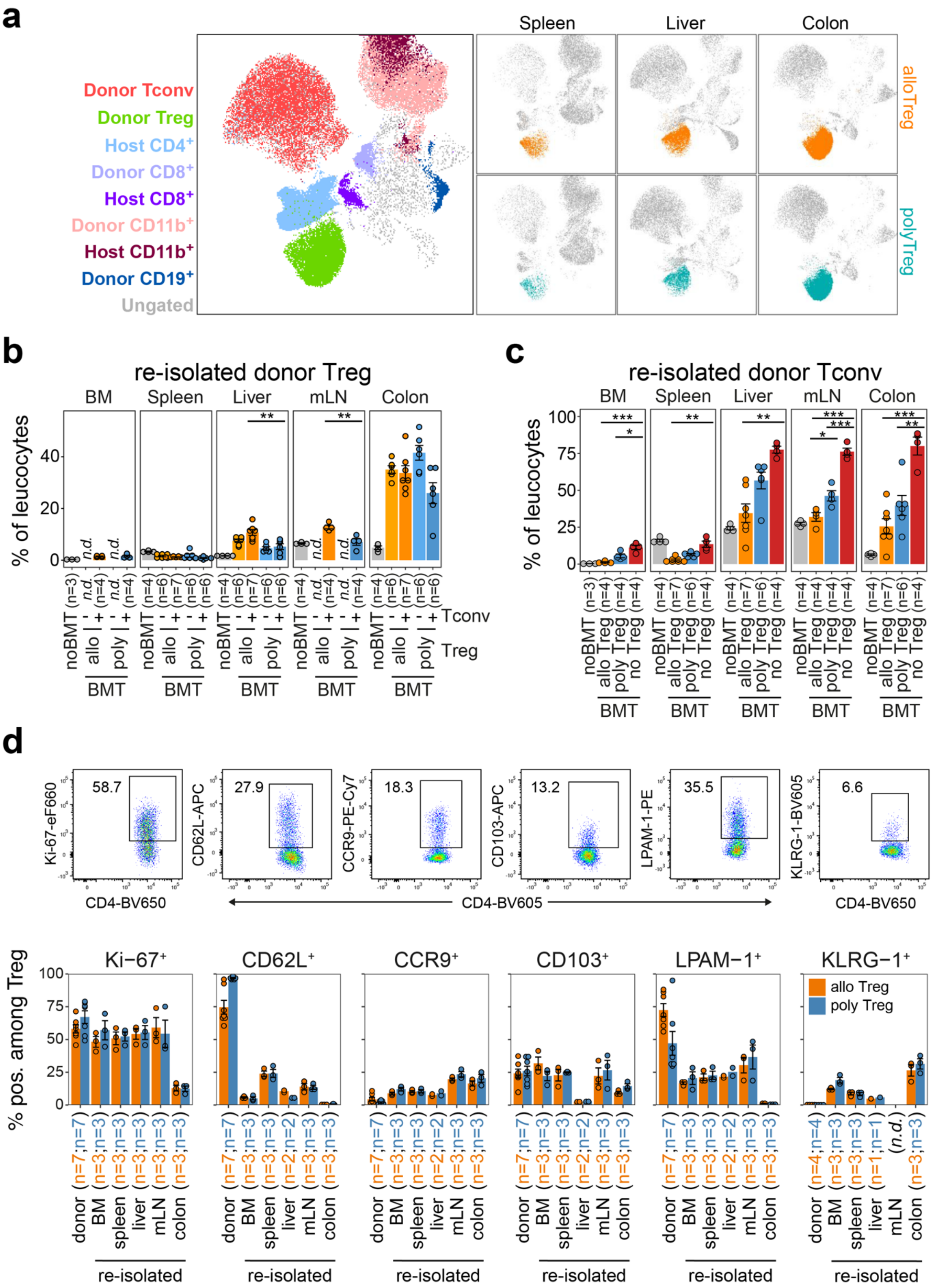

(continued on next page)

Supplemental Figure 2 (continued)

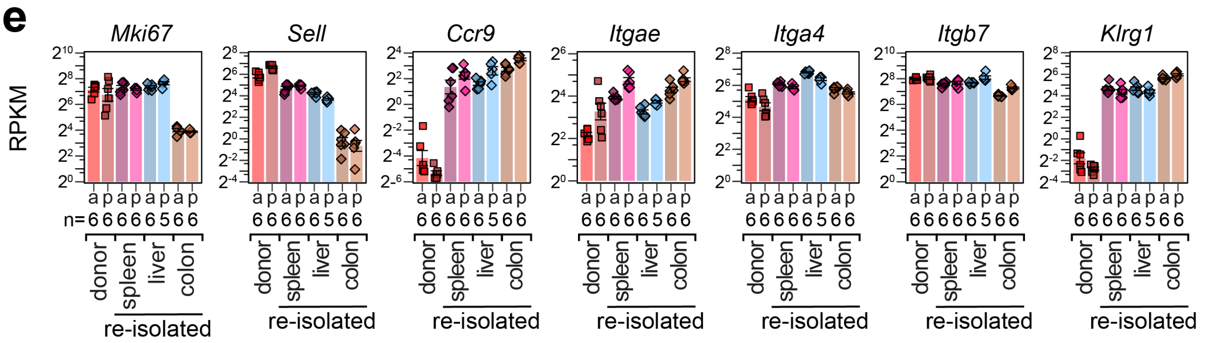

Donor Treg migrate into non-lymphoid tissues 7 days after allogeneic BMT, Related to Figure 2. (a) UMAP embedding of live CD45.1+TER119- and CD45.2+TER119- single cell suspensions re-isolated from recipient animals on day 7 after BMT, as analyzed by multiparametric flow cytometry. Data from 27 samples, representing cell pools of 2-3 animals each, originating from 3 different groups (allo and polyTreg prophylaxis and GvHD), and 3 independent experiments were combined. Large panel: all data, all major donor and host cell populations are highlighted according to the colored legend on the left. Smaller panels: data stratified by organ and expansion method (allo/poly), only donor Treg are colored. (b) Bar plots showing (donor) Treg cell numbers relative to total leukocyte counts isolated from the respective organs of non-transplanted donor mice (noBMT) or transplanted recipient mice (BMT) that received allo or polyTreg together with TCDBM (no GvHD) or additional Tconv (prophylaxis). (c) Bar plots showing Tconv cell numbers relative to total leukocyte counts isolated from the respective organs of non-transplanted donor mice (noBMT) or transplanted recipient mice (BMT) that received TCDBM and Tconv either together with allo or polyTreg (prophylaxis) or w/o Treg (GvHD). (b,c) Bars represent mean values ±SEM and dots represent data from individual recipients. Significant differences between both BMT groups are indicated above bars. (1-way ANOVA with Tukey's post hoc, except: BM and mLN in (b), 2-tailed t-test; spleen and liver in (c), Kruskal-Wallis test with Dunn's post hoc) * $P < 0.05$, ** $P < 0.01$, *** $P < 0.001$. (d) Gating for and flow cytometric analysis of indicated homing and activation markers in donor allo and polyTreg either directly after *in vitro* expansion and before injection ('donor') or after re-isolation from indicated organs 7d after transfer into MHC-disparate hosts. Top panels show marker gates in representative plots of re-isolated donor Treg. Bottom panels show combined data from n=7 independent cultures and n=3 individual transplant experiments. Bars represent mean values ± SEM, dots represent individual data points. (e) Expression profiles of genes corresponding to FACS markers. Note that the expression of integrin chain genes may vary from surface expression of integrin dimers. Bars represent mean±SEM of RPKM values from n=4-6 independent experiments. Individual data points are shown as dots. (b,c,d,e) Source data and exact P values (b,c) are provided as a Source Data file.

Supplemental Figure 3

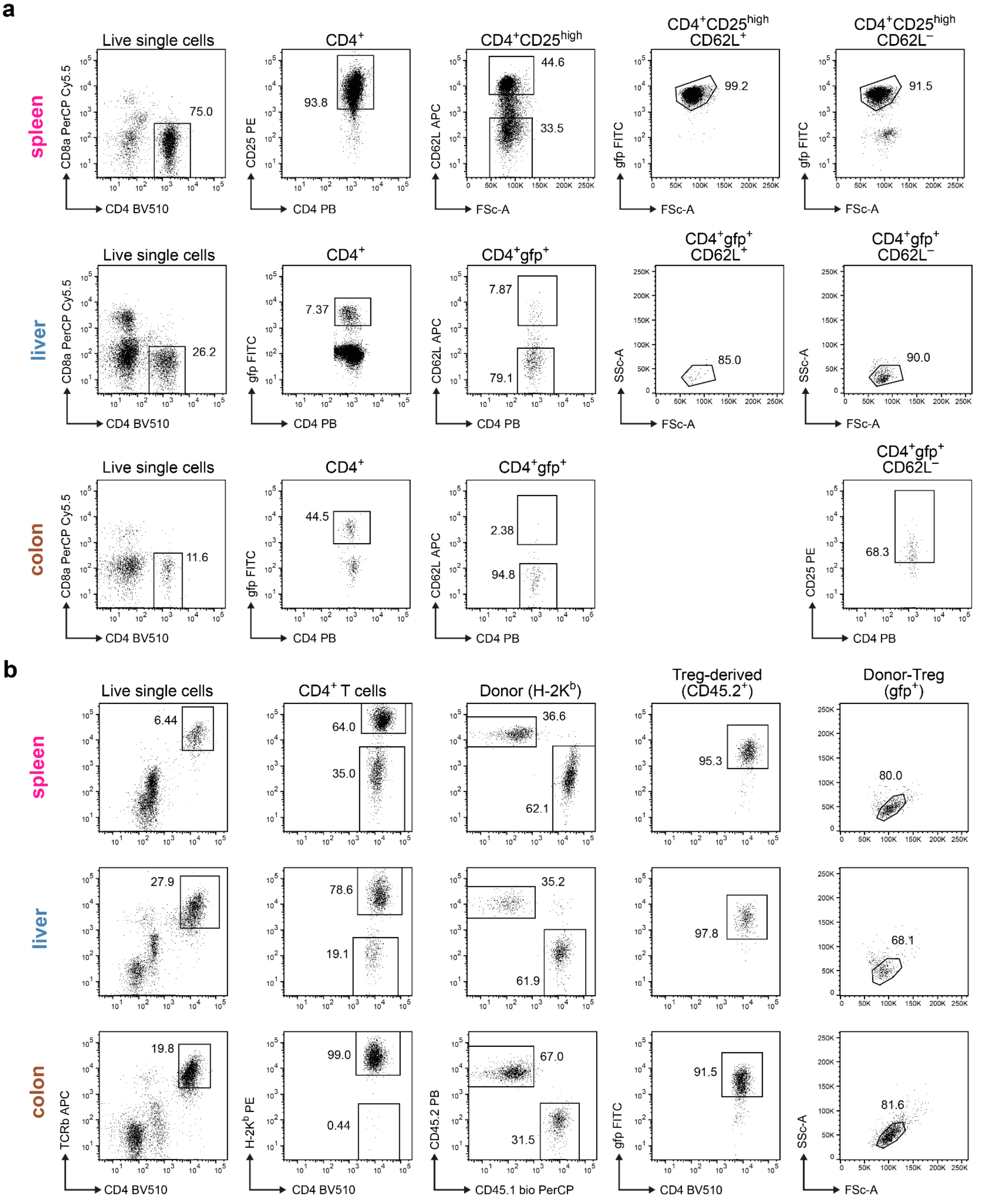

(continued on next page)

Supplemental Figure 3 (continued)

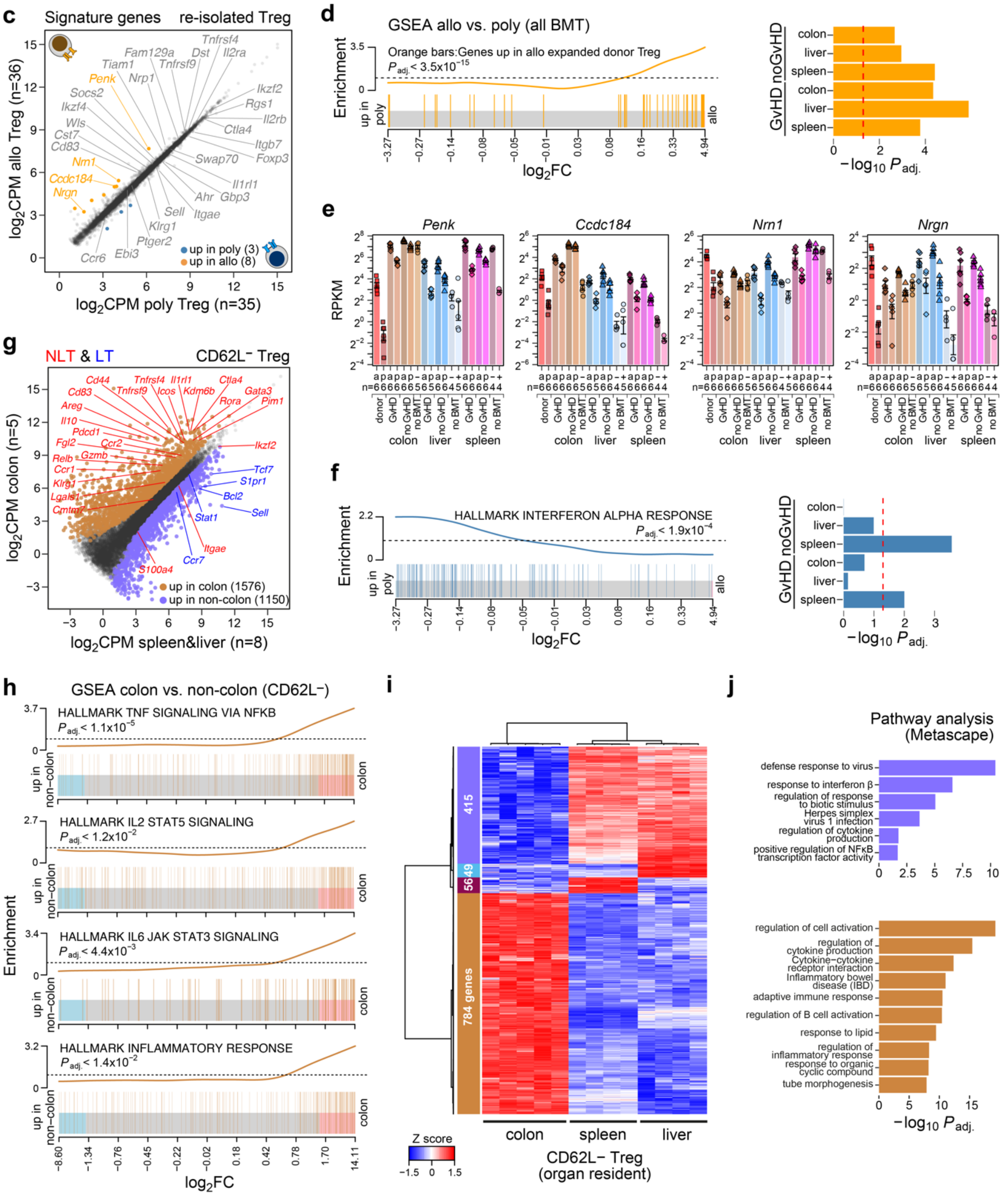

(continued on next page)

Supplemental Figure 3 (continued)

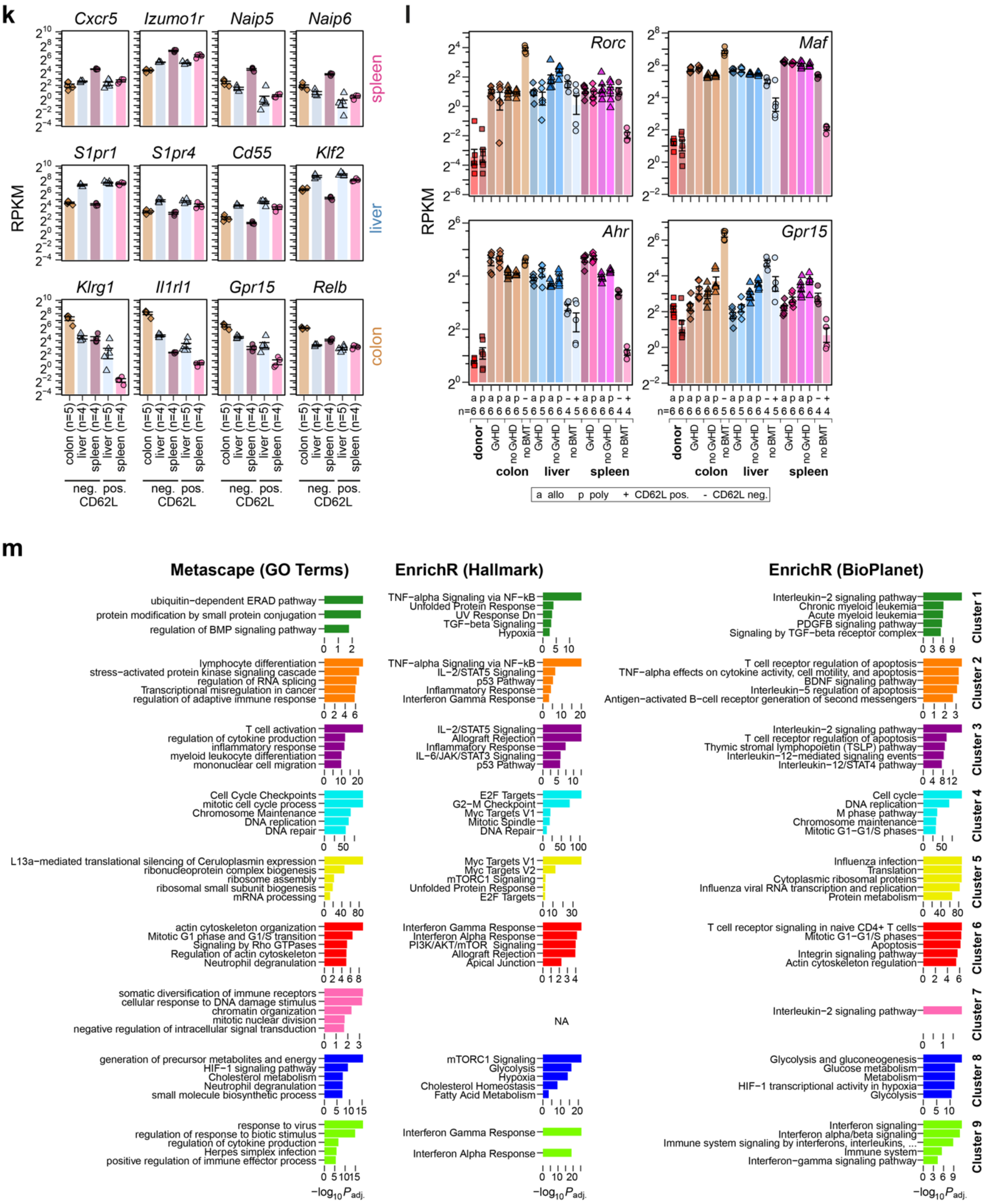

(continued on next page)

Supplemental Figure 3 (continued)

Treg gene expression profiles of organ-resident Treg and donor Treg re-isolated from MHC-disparate BMT recipients, Related to Figure 3. (a) Gating strategy for the sorting of tissue-resident Treg from naïve FoxP3^{gfp} mouse organs (noBMT). Representative plots of live single cells are shown for sorting of CD4^{+}CD25^{high}CD62L^{+}gfp^{+} and CD4^{+}CD25^{high}CD62L^{-}gfp^{+} Treg from CD25-pre-enriched splenocytes, or leukocytes isolated from liver or colon. Colonic cells were additionally pre-gated on CD45. (b) Gating strategy for the sorting of Tregs re-isolated from spleen, liver and colon of BALB/c recipients, seven days after BMT (as detailed in Fig. 2). Representative plots are shown for the gating strategy of TCRβ^{+}CD4^{+}CD45.2^{+}gfp^{+} Treg in all three organs. (c) Scatter plot of global gene expression (log_2CPM>1, log_2RPKM>1) comparing donor allo and polyTreg re-isolated from BMT recipients. Differentially expressed genes are highlighted by coloring ($P_{adj.|FC|>1.5}$ >0.05, qlf-test). Treg-signature genes as defined in Fig.1e and not significantly different between allo and polyTreg are labeled in grey. (d) Barcode plot presenting GSEA results for the indicated gene list across the logFC ranking of genes based on the comparison of allo and polyTreg across all BMT samples. Enrichment *P* values of two-sided rotation gene set tests are given for all samples (left panel) or for condition/organ-wise comparisons (bar plot, right panel). (e) Examples for expression profiles of genes retaining poly-allo expression differences *in vivo*. Bars represent means±SEM of RPKM values from independent experiments. Individual data points are shown as dots and their numbers are indicated in the axis labels. (f) Barcode plot presenting GSEA results for the indicated Hallmark gene set across the logFC ranking of genes based on the comparison of allo and poly Treg. Benjamini-Hochberg adjusted enrichment *P* values of competitive gene set tests are given for all samples (left panel) or for condition/organ-wise comparisons (bar plot, right panel). The dashed red lines in bar plots in (d,f) mark the FDR value of 0.05. (g) Scatter plot of global gene expression comparing organ-resident (no BMT) CD62L^{-} Treg isolated from colon versus non-colon samples. Differential genes are highlighted by coloring ($P_{adj.|FC|>1.5}$ >0.05, qlf-test). Labels indicate NLT (red) and LT (blue) signature genes as defined by Miragaia et al. [1]. (h) Barcode plots presenting GSEA results for the indicated Hallmark gene set across the logFC ranking of genes based on the comparison of organ-resident (no BMT) CD62L^{-} colon versus non-colon Treg. Benjamini-Hochberg adjusted enrichment *P* values of competitive gene set tests are given. (i) Heatmap presenting hierarchically clustered and scaled expression data of genes that were differential between Treg isolated from the three indicated organs (fold-change > 2 & FDR < 0.05, log_2CPM>1, log_2RPKM>1). Clusters are color-coded and sizes are indicated on the left. (j) For clusters shown in (i), the leading enriched terms or pathways (as analyzed using Metascape) are given. For the (purple) spleen-specific cluster or the turquoise liver-specific cluster, no enriched pathways were found. (k) Examples of organ-specific gene expression profiles. Bars represent means±SEM of RPKM values from independent experiments. Individual data points are shown as dots and their numbers are indicated in the axis labels. (l) Expression profiles of markers of peripherally-induced Treg. Bars represent means±SEM of RPKM values from independent experiments. Individual data points are shown as dots and their numbers are indicated in the axis labels. (m) For co-expression clusters shown in Fig. 3d, the leading enriched terms or pathways (as analyzed using Metascape and EnrichR) are given. (c-m) Source data are provided as a Source Data file.

Supplemental Figure 4

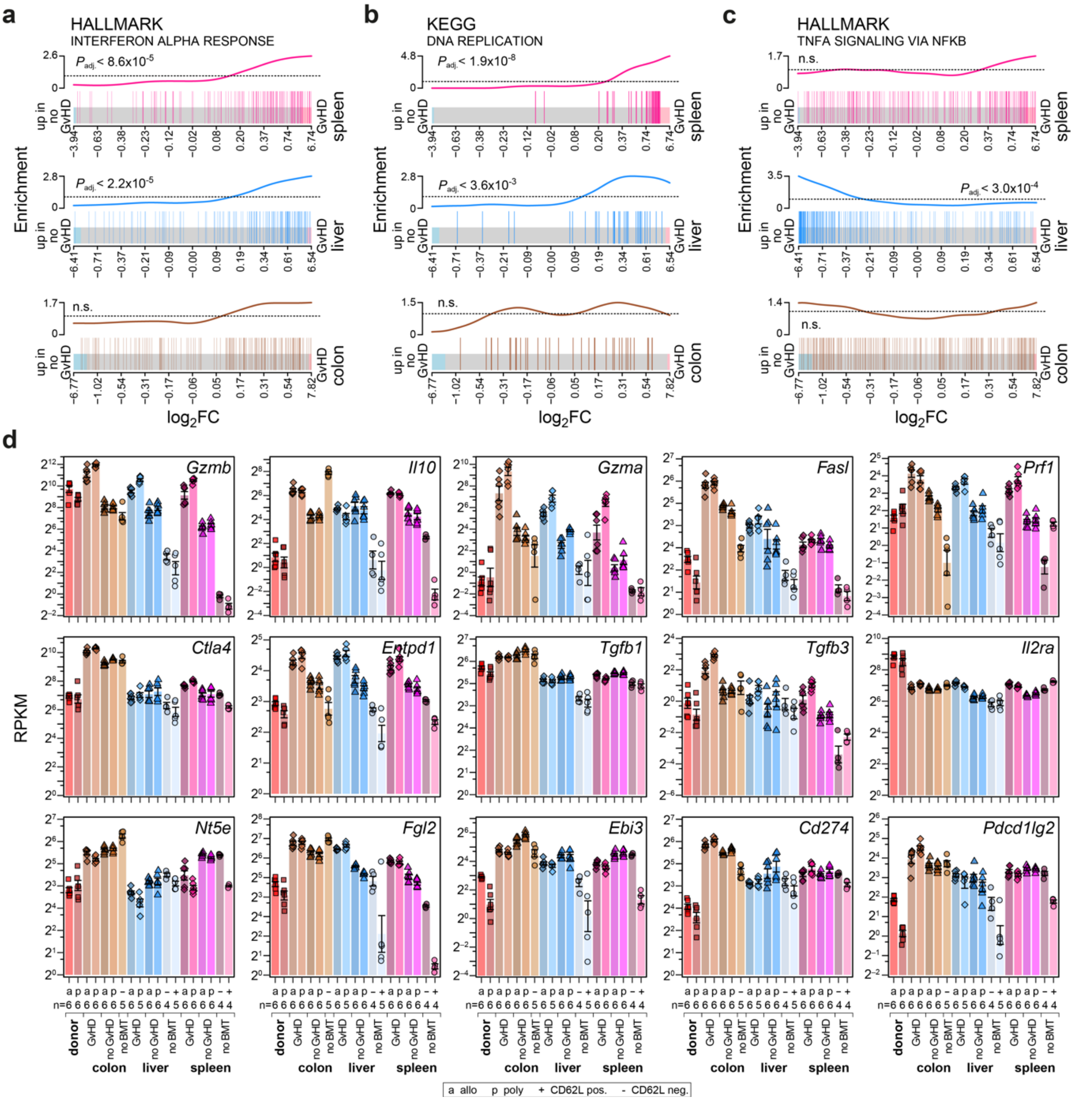

GvHD induced gene expression programs in organ-infiltrating donor Tregs, Related to Figure 4. (a-c) Barcode plots presenting GSEA results for the indicated Hallmark and Kegg gene sets across the logFC ranking of genes based on the comparison of re-isolated donor Treg from the indicated organs after BMT +/- Tconv (GvHD versus no GvHD). Benjamini-Hochberg adjusted enrichment *P* values of competitive gene set tests are given. n.s., not significant (d) Expression profiles of genes associated with suppressive Treg functions. Bars represent means±SEM of RPKM values from independent experiments. Individual data points are shown as dots and their numbers are indicated in the axis labels. (d) Source data are provided as a Source Data file.

Supplemental Figure 5

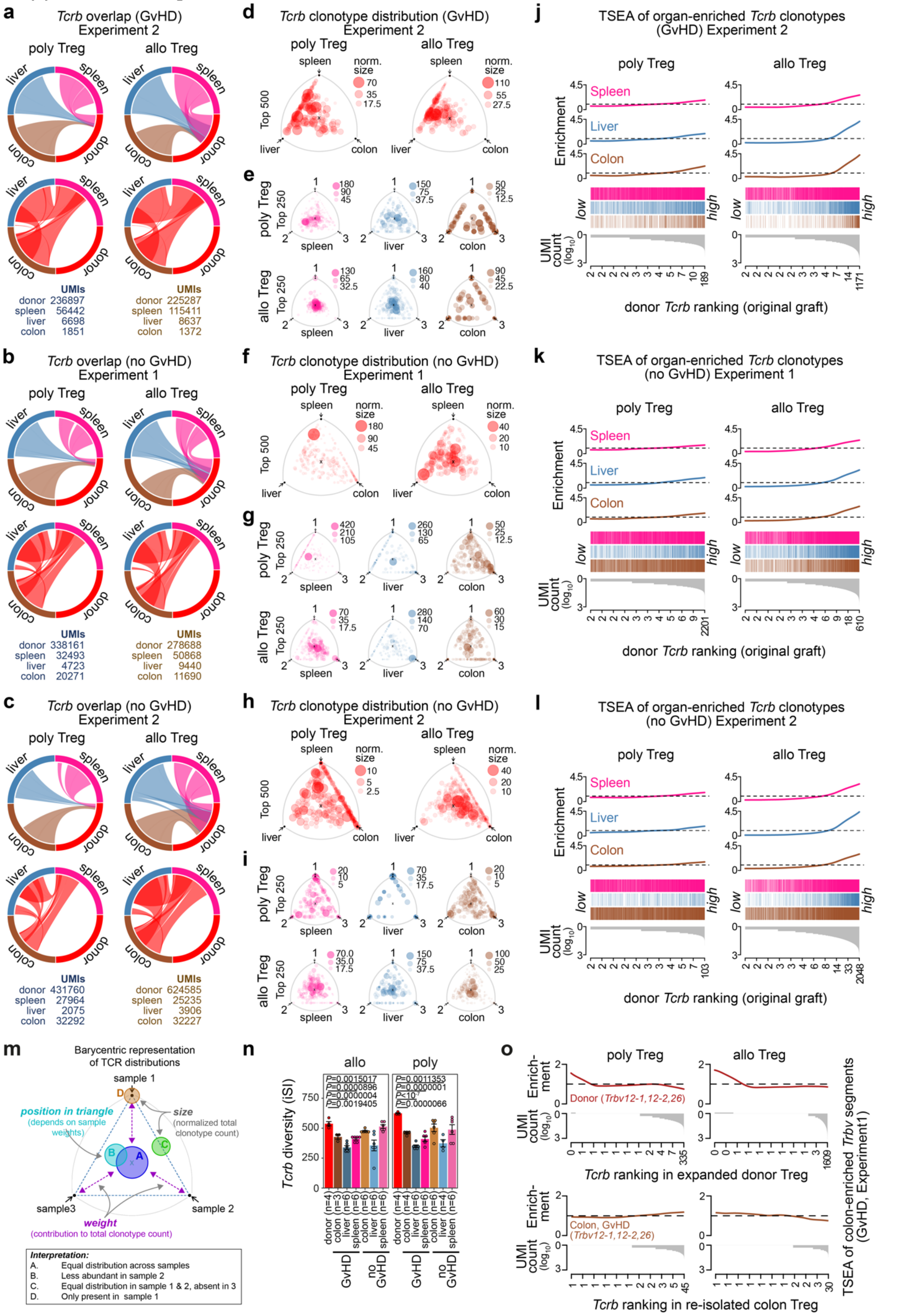

(continued on next page)

Supplemental Figure 5 (continued)

Clonotypes of re-isolated Treg, Related to Figure 5. (a-c) Circos plots presenting the donor Treg TCR repertoire overlap in the prophylaxis set-up (GvHD, in a) and the control BMT without Tconv (no GvHD, in b,c) between graft (donor, poly or allo) and recipient organs (top panels) or between organs (bottom panels) across recipients of the same donor Treg product. Bands represent the fractions of UMI overlaps of pooled organs. Total numbers of *Tcrb* UMIs (representing single mRNA molecules) are given below the plots. (d,f,h) Corresponding barycentric distributions of *Tcrb* clonotypes between organs (recipients of the same graft were merged). (e,g,i) Barycentric distributions of *Tcrb* clonotypes between replicates of individual organs. (j,k,l) TCR set enrichment analysis (TSEA) of donor Treg *Tcrb* clonotypes detected in host organs after BMT (prophylaxis) across frequency-ranked clonotypes of the originally grafted poly or allo donor Treg products. (m) Schematic presentation and interpretation of barycentric triangle plots. The position of a bubble in the triangle reflects the respective organ contribution to the clonotype and bubble size corresponds to the normalized size of each clone. (n) Barplot of *Tcrb* clonotype diversities (iSI, inverse Simpson index) across allo and poly expanded Treg (donor) and donor Treg re-isolated from the indicated organs. Bars represent the means±SEM of independent experiments. Individual data points are shown as dots and their numbers are indicated in the axis labels. Significant differences between groups are indicated above bars (1-way ANOVA with Tukey's post hoc test). To avoid bias due to differences in cell counts, repertoires were downsampled to the sample with the lowest count (645 TCR clones). (o) TSEA of donor Treg *Tcrb* clonotypes with colon-enriched *Trbv* segments across frequency-ranked clonotypes of the originally grafted donor poly or allo Treg products (left panels) or donor Tregs reisolated from colon (right panels). (a-l,n) Source data are provided as a Source Data file.

Supplemental Figure 6

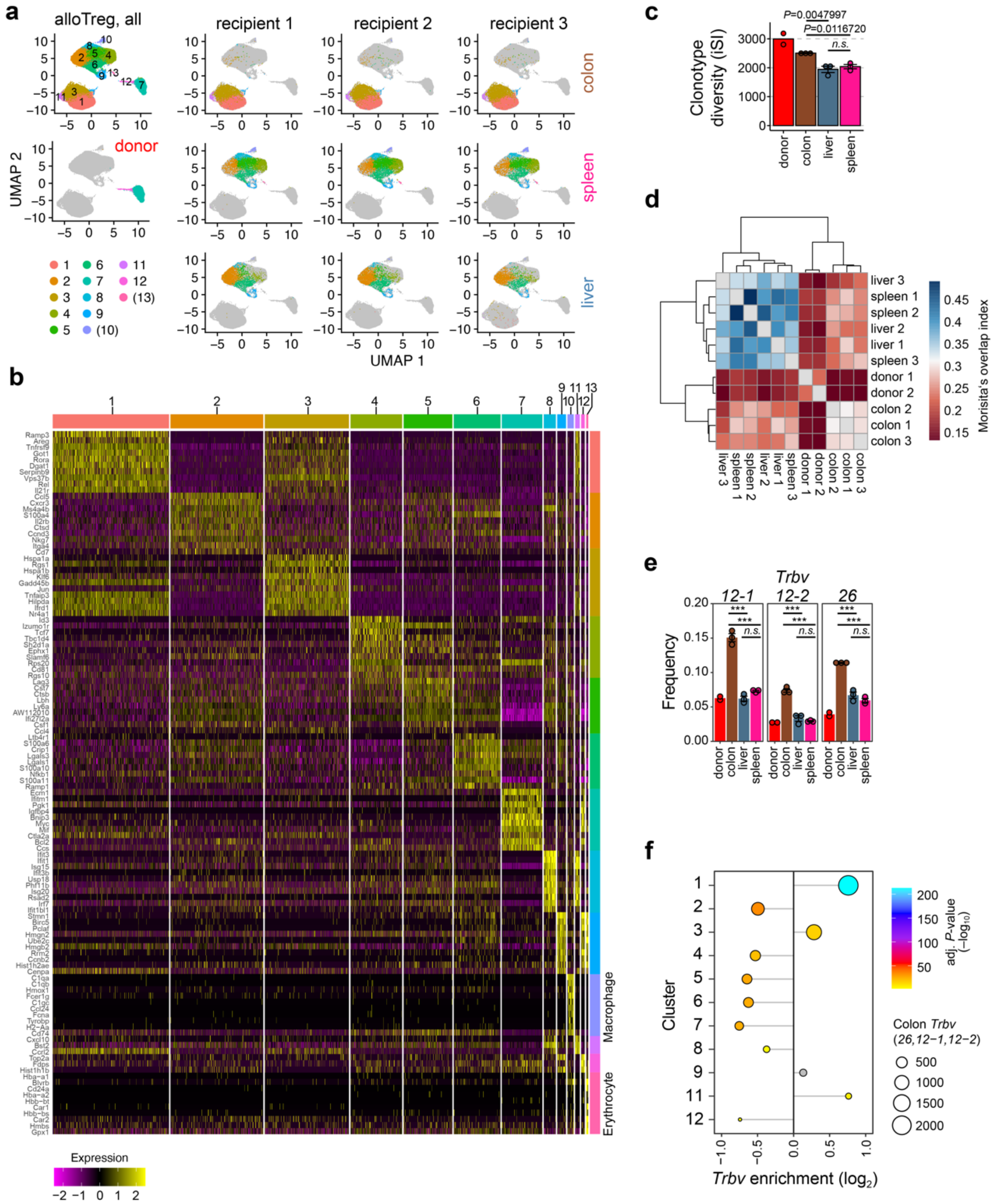

TCR-independent transcriptional re-wiring of donor Treg in recipient organs after BMT, Related to Figure 6. (a) Distribution of individual samples in the UMAP embedding shown in Figure 6a. Original clusters are colored as indicated. (b) Heatmap of top marker genes across clusters 1-13. (c) Barplot of TCR diversities (iSI, inverse Simpson index) in the donor Treg populations (n=2 technical replicates; bar represents the mean) or donor Treg re-isolated from spleen, colon, and liver (n=3 individual recipients of the same graft; bars represent means±SEM). Individual data points are shown as dots. Significant differences between tissue are indicated above bars (1-way ANOVA with Tukey's post hoc test). (d) Hierarchically clustered heatmap of Morisita's overlap indices for the indicated samples. (e) Frequencies of three colon-enriched *Trbv* segments across allo expanded Treg (donor) and donor Treg re-isolated from the indicated organs (as shown in c). Significant differences between tissue are indicated above bars (1-way ANOVA with Tukey's post hoc test). (f) Enrichment of the three *Trbv* segments (*Trbv12-1, 12-2, 26*) compared to all other *Trbv* segments across sub-clusters 1-9 & 11,12 (two-sided Fisher's exact test with Benjamini-Hochberg correction). (e) * $P < 0.05$, ** $P < 0.01$, *** $P < 0.001$. (b-f) Source data and exact P values (e) are provided as a Source Data file.

Supplemental Figure 7

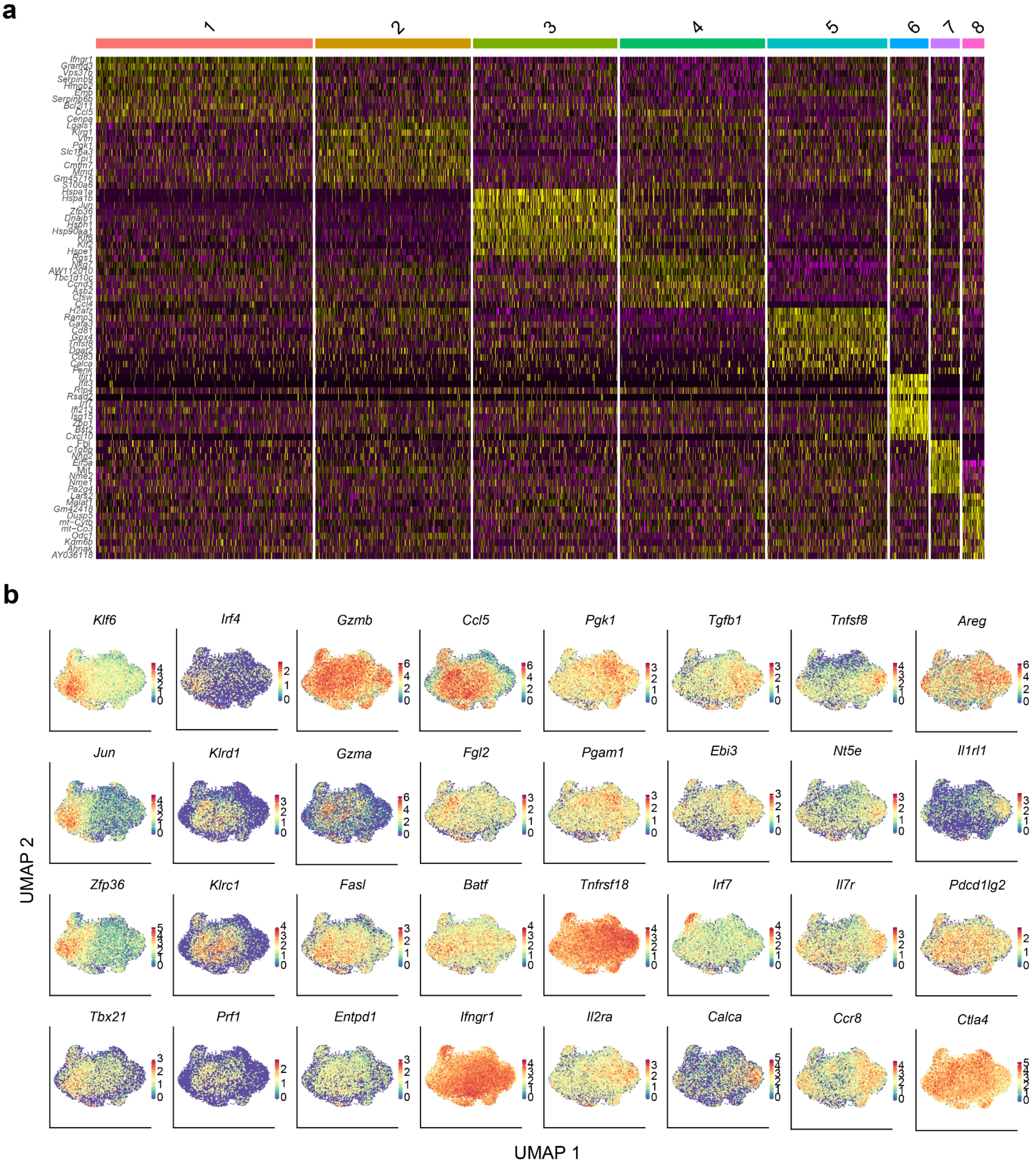

Colon-specific reprogramming of donor Treg after BMT, Related to Figure 7. (a) Heatmap of top marker genes across colon clusters 1-8. (b). Expression of selected marker genes across the UMAP embedding. Expression levels are in log2 scale and color coded as shown in the corresponding legends. (a) Source data are provided as a Source Data file.

Supplemental Table 1
Signature gene lists

List	Figure	Genes	Reference
Treg signature	1e,f; S3a	*Ahr, Arhgap20, Ccr6, Cd83, Cst7, Ctla4, Dst, Ebi3, Fam129a, Foxp3, Gbp3, Gpr83, Ikzf2, Ikzf4, Il1rl1, Il2ra, Il2rb, Itgae, Klrg1, Nrp1, Penk, Plagl1, Prg4, Ptger2, Rgs1, Socs2, Swap70, Tiam1, Tnfrsf4, Tnfrsf9, Wls, Itgb7, Sell*	[2]
Lymphoid tissue (LT) markers	3b, S3e	*Tcf7, Bcl2, Sell, S1pr1, Ccr7, Stat1*	[1]
Non-lymphoid tissue (NLT) markers	3b, S3e	*Tnfrsf4, Tnfrsf9, Relb, Cd83, Pdcd1, Ikzf2, Pim1, Ctla4, Icos, Cd44, Cmtm7, Fgl2, Lgals1, Klrg1, Rora, Itgae, S100a4, Areg, Gata3, Kdm6b, Il1rl1, Il10, Gzmb, Ccr2, Ccr1*	[1]
Suppression by Treg	4a-d	*Cd274, Ctla4, Ebi3, Entpd1, Fasl, Fgl2, Gzma, Gzmb, Il10, Il2ra, Nt5e, Pdcd1lg2, Prf1, Tgfb1, Tgfb3*	[3, 4, 5, 6]
peripherally induced Treg	S4e	*Ahr, Gpr15, Maf, Rorc*	[7]
Interferon response	7c	*Ifit1, Ifit3, Ifi208, Ifi214, Ifit3b, Irf7, Ifi213, Rsad2, Rtp4, Cmpk2*	[8]
TNF signaling via NFκB	7c	*Jun, Zfp36, Klf6, Klf2, Tnfrsf9, Ppp1r15a, Nfkbia, Junb, Dusp1, Ier2, Cd69, Nr4a1, Fos, Btg2, Gadd45b, Tnfaip3, Atf3, Irf1, Map3k8, Gpr183, Btg1*	[8]
Glycolysis	7c	*Hspa5, Slc16a3, Aldoa, Pgk1, Tpi1, Ldha, Pkm, Pgam1, Got1, Glrx, Eno1*	[8]
Myc targets	7c	*Fbl, Apex1, C1qbp, Nhp2, Nop16, Rps2, Npm1, Nme1, Eif4a1, Pa2g4, Ppia, Ranbp1, Mrps18b, Nolc1, Phb, Set, Ran, Snrpd3, Hsp90ab1, Cct5, Ddx21, Abce1, Ldha, Nop56, Cnbp, Impdh2, Ruvbl2, Cct3, Lsm7, Hnrnpa1, Gnl3, Mrpl23, Snrpa1, Eif3b, Snrpa, Serbp1, Eif2s1, Rack1, Tcp1, Cct2, Ddx18, Hspd1, Slc25a3, G3bp1, Rpl14, Phb2, Hnrnpa2b1, Eif4e, Tomm70a, Hspe1, Snrpd1, Cdk4, Prpf31, Syncrip, U2af1, Cyc1, Eif1ax, Eif3d, Snrpd2, Lsm2, Pgk1, Rps6, Psma7, Ndufab1, Ube2e1, Canx*	[8]

Supplemental Table 2
Antibodies used for flow cytometry

ANTIGEN	CLONE	DILUTIONS/ AMOUNTS	FLUORO-CHROME	SOURCE	ORDER CODE
**BIOTIN**	REA746	1:50	PerCP	Miltenyi Biotec	130-110-960
**CD4**	RM4-5	1:200<br>1:100<br>1:133.3	PB<br>BV510<br>BV605	BD Biosciences	558107<br>563106<br>563151
**CD8A**	53-6.7	1:100<br>1: 00	BUV805<br>PerCP-Cy5-5	BD Biosciences	612898<br>551162
**CD11B**	M1/70	1:1000<br>1:200	PE<br>PE-Cy7	eBioscience<br>BD Biosciences	12-0112-82<br>552850
**CD11C**	N418	1:100	PerCP	BioLegend	117326
**CD16/32**	93	1:50	Purified	BioLegend	101302
**CD19**	1D3	1:133.3<br>1:133.3	BV650<br>FITC	BD Biosciences	563235<br>553785
**CD25**	PC61	1:100	PE	BD Biosciences	553866
**CD25**	PC61.5	1:200	PE-Cy7	eBioscience	25-0251-82
**CD45**	30F-11	1:200	BV510	BD Biosciences	563891
**CD45.1**	A20	1:100<br>1:200	BUV395<br>Biotin	BD Biosciences<br>BioLegend	565212<br>110704
**CD45.2**	104	1:200<br>1:100	PB<br>BV786	BioLegend<br>BD Biosciences	109820<br>563686
**CD62L**	MEL-14	1:133.3	APC	BD Biosciences	553152
**CD103**	2E7	1:80	APC	eBioscience	17-1031-82
**CD199/CCR9**	eBioCW-1.2	1:200	PE-Cy7	eBioscience	17-1991-82
**FOXP3**	FJK-16s	1:100	FITC	eBioscience	11-5773-82
**GR-1**	RB6-8C5	1:200	APC	BD Biosciences	553129
**H-2K^b**	AF6-88.5	1:100<br>1:100	Biotin<br>PE	BioLegend	116504<br>116508
**HELIOS**	22F6	1:20	PE	BioLegend	137216
**KI-67**	SolA15	1:2000	eFluor660	eBioscience	50-5698-82
**LY6C**	HK1.4	1:100	PB	BioLegend	128014
**KLRG-1**	2F1/KLRG1	100	BV605	BioLegend	138419
**LPAM-1**	DATK32	1:80	PE	BD Biosciences	553811
**NKP46**	29A1.4	1:25	BV510	BD Biosciences	563455
**TCRβ**	H57-597	1:200<br>1:50	PE<br>BUV737	BD Biosciences	553172<br>612821
**TCRβ**	REA318	1:20	APC	Miltenyi Biotec	130-104-810
**TER-119**	TER-119	1:100	APC-eF780	eBioscience	47-5921-82

Supplemental Table 3
Oligonucleotides used for TCR-seq

Name	Species	Chain	Type	i5/i7 index	Sequence 5'→3' [a]
5'CDS Primer	universal	universal	cDNA synthesis		TTTTTTTTTTTTTTTTTTTTTTTTTVN
TCR_UMI_TSO	universal	universal	cDNA synthesis		Bio-GUCUCGUGGGCUCGGAGAUGUGUAUAAGAGACAGUNNNNUNNNNUNNNNUrGrGrG
TCR_1.PCR_for	universal	universal	1st PCR		GTCTCGTGGGCTCGGAGATGTG
GSP_TRA_rev	Mm	α	1st PCR		GTCGGTGAACAGGCAGAGGGT
GSP_TRB_rev	Mm	β	1st PCR		GAAGCCCCTGGCCAAGCACACGAG
Primer_i7 (for)	universal	universal	2nd PCR	701-729	CAAGCAGAAGACGGCATACGAGATXXXXXXXXGTCTCGTGGGCTCGG
TRA_2nd_rev01	Mm	α	2nd PCR	501	AATGATACGGCGACCACCGAGATCTACACTAGATCGCTCGTCGGCAGCGTCAGATGTGTATAAGAGACAGGATCTTTTAACTGGTACACAGCA
TRA_2nd_rev02	Mm	α	2nd PCR	502	AATGATACGGCGACCACCGAGATCTACACCTCTCTATTCGTCGGCAGCGTCAGATGTGTATAAGAGACAGGATCTTTTAACTGGTACACAGCA
TRA_2nd_rev03	Mm	α	2nd PCR	503	AATGATACGGCGACCACCGAGATCTACACTATCCTCTTCGTCGGCAGCGTCAGATGTGTATAAGAGACAGGATCTTTTAACTGGTACACAGCA
TRA_2nd_rev08	Mm	α	2nd PCR	508	AATGATACGGCGACCACCGAGATCTACACCTAAGCCTTCGTCGGCAGCGTCAGATGTGTATAAGAGACAGGATCTTTTAACTGGTACACAGCA
TRB_2nd_rev01	Mm	β	2nd PCR	501	AATGATACGGCGACCACCGAGATCTACACTAGATCGCTCGTCGGCAGCGTCAGATGTGTATAAGAGACAGTTGATGGCTCAAACAAGGAG
TRB_2nd_rev02	Mm	β	2nd PCR	502	AATGATACGGCGACCACCGAGATCTACACCTCTCTATTCGTCGGCAGCGTCAGATGTGTATAAGAGACAGTTGATGGCTCAAACAAGGAG
TRB_2nd_rev17	Mm	β	2nd PCR	517	AATGATACGGCGACCACCGAGATCTACACGCGTAAGATCGTCGGCAGCGTCAGATGTGTATAAGAGACAGTTGATGGCTCAAACAAGGAG
TRB_2nd_rev20	Mm	β	2nd PCR	520	AATGATACGGCGACCACCGAGATCTACACAAGGCTATTCGTCGGCAGCGTCAGATGTGTATAAGAGACAGTTGATGGCTCAAACAAGGAG

[a] Bio- = 5' biotinylated, U = deoxyuridine, rG = ribo-Guanosin

Supplemental Table 4
RNA- and TCR-sequencing data of Treg donor samples (accession IDs: RNA GSE223796, TCR GSE223797)

Name	Culture Time (in d)	Expansion Type	Cell Count (RNA)	RIN[1]	Transcriptome (Unique Reads[2])	*Tcra* MIGs[3]	*Tcrb* MIGs
**alloTreg_1_donor**	14d	allo	1000000	7	N/A	137475	144229
**alloTreg_2_donor**	13d	allo	3000000	10	11738045	363756	120366
**alloTreg_3_donor**	12d	allo	3000000	9.6	11959184	416994	350186
**alloTreg_4_donor**	11d	allo	12600000	7.9	10608848	225577	539682
**alloTreg_5_donor**	11d	allo	3000000	9.5	16105016	N/A	N/A
**alloTreg_6_donor**	12d	allo	10000000	8.4	15823572	83357	278688
**alloTreg_7_donor**	12d	allo	10000000	9.8	14070458	343000	624585
**polyTreg_1_donor**	14d	poly	800000	10	N/A	331293	75320
**polyTreg_2_donor**	13d	poly	3000000	10	11586946	363945	236897
**polyTreg_3_donor**	12d	poly	3000000	9.6	11621484	331739	298095
**polyTreg_4_donor**	11d	poly	6000000	9.2	14073418	298946	579305
**polyTreg_5_donor**	11d	poly	6000000	9.7	13579460	N/A	N/A
**polyTreg_6_donor**	12d	poly	10000000	9	15401376	117378	338161
**polyTreg_7_donor**	12d	poly	10000000	9.8	14405457	287403	431760

[1]RNA-Integrity Index (RIN) as measured using the TapeStation (Agilent)
[2]Unique sequences after mapping to GRCm38
[3]MIG: Molecular identifier group = number of UMIs

Supplemental Table 5
RNA- and TCR-sequencing data of organ-resident Treg (accession IDs: RNA GSE223796, TCR GSE223797)

Name	Source	CD62L stain	Cell Count (RNA)	RIN	Transcriptome (Unique Reads)	*Tcra* MIGs	*Tcrb* MIGs
**colonTreg_1_resident**	colon	neg.	53000	N/A	16688735	N/A	N/A
**colonTreg_2_resident**	colon	neg.	8188	N/A	15357437	268	319
**colonTreg_3_resident**	colon	neg.	6000	N/A	9998437	293	310
**colonTreg_4_resident**	colon	neg.	8699	N/A	13466513	151	236
**colonTreg_5_resident**	colon	neg.	10700	N/A	14697092	270	266
**liverCD62LnegTreg_1_resident**	liver	neg.	14000	6.8	14495223	605	1497
**liverCD62LnegTreg_2_resident**	liver	neg.	31400	N/A	11085535	597	1560
**liverCD62LnegTreg_3_resident**	liver	neg.	33000	N/A	12501314	737	1677
**liverCD62LnegTreg_4_resident**	liver	neg.	53000	N/A	16325268	3567	6570
**liverCD62LposTreg_1_resident**	liver	pos.	8200	N/A	13033678	125	338
**liverCD62LposTreg_2_resident**	liver	pos.	9500	N/A	11323276	25	101
**liverCD62LposTreg_3_resident**	liver	pos.	4300	7.9	11511117	N/A	N/A
**liverCD62LposTreg_4_resident**	liver	pos.	8400	N/A	11218895	138	390
**liverCD62LposTreg_5_resident**	liver	pos.	3400	N/A	9907473	84	238
**spleenCD62LnegTreg_1_resident**	spleen	neg.	1400000	9.4	16462734	170204	339958
**spleenCD62LnegTreg_2_resident**	spleen	neg.	960000	9.3	11236636	185448	346006
**spleenCD62LnegTreg_3_resident**	spleen	neg.	600000	8.5	15622731	125175	234498
**spleenCD62LnegTreg_4_resident**	spleen	neg.	497000	N/A	13176198	28009	55252
**spleenCD62LposTreg_1_resident**	spleen	pos.	1200000	9.4	17802510	203742	214328
**spleenCD62LposTreg_2_resident**	spleen	pos.	900000	8.8	14721974	149086	183849
**spleenCD62LposTreg_3_resident**	spleen	pos.	700000	8.1	15145484	142178	223493
**spleenCD62LposTreg_4_resident**	spleen	pos.	779000	N/A	14194111	30251	73244
**spleenCD62LposTreg_5_resident**	spleen	pos.	1000000	8.7	N/A	154132	33059
**spleenCD62LposTreg_6_resident**	spleen	pos.	1000000	9.5	N/A	33033	91049

Supplemental Table 6
RNA- and TCR-sequencing data of re-isolated Treg – control BMTs (no GvHD)
(accession IDs: RNA GSE223796, TCR GSE223797)

Name	Source	BMT Donor	Recipient	Cell Count (RNA)	RIN	Transcriptome (Unique Reads)	*Tcra* MIGs	*Tcrb* MIGs
**colonTreg_6-1_noGvHD**	colon	alloTreg_6	rec-6-1	17000	8.8	14328979	4795	5827
**colonTreg_6-2_noGvHD**	colon	alloTreg_6	rec-6-2	86000	8.2	14927471	2869	3956
**colonTreg_6-3_noGvHD**	colon	alloTreg_6	rec-6-3	62000	N/A	15078806	1367	1572
**colonTreg_7-1_noGvHD**	colon	alloTreg_7	rec-7-1	55000	9.7	15847530	9126	9500
**colonTreg_7-2_noGvHD**	colon	alloTreg_7	rec-7-2	52000	9.5	14458588	10027	10158
**colonTreg_7-3_noGvHD**	colon	alloTreg_7	rec-7-3	55000	9.6	14792209	13355	12569
**colonTreg_6-4_noGvHD**	colon	polyTreg_6	rec-6-4	4600	9.1	14975720	7883	9173
**colonTreg_6-5_noGvHD**	colon	polyTreg_6	rec-6-5	38000	7.9	15152912	4780	5302
**colonTreg_6-6_noGvHD**	colon	polyTreg_6	rec-6-6	35000	8.7	13778207	4686	5796
**colonTreg_7-4_noGvHD**	colon	polyTreg_7	rec-7-4	53000	9.6	13507640	13843	15060
**colonTreg_7-5_noGvHD**	colon	polyTreg_7	rec-7-5	47000	9.6	15344469	6823	7060
**colonTreg_7-6_noGvHD**	colon	polyTreg_7	rec-7-6	60000	9.6	13379430	8978	10172
**liverTreg_6-1_noGvHD**	liver	alloTreg_6	rec-6-1	4600	N/A	14787156	1123	1754
**liverTreg_6-2_noGvHD**	liver	alloTreg_6	rec-6-2	11600	7.3	14943012	2379	4212
**liverTreg_6-3_noGvHD**	liver	alloTreg_6	rec-6-3	7900	6.7	15124724	1045	2814
**liverTreg_7-1_noGvHD**	liver	alloTreg_7	rec-7-1	7000	N/A	15406247	1320	2039
**liverTreg_7-2_noGvHD**	liver	alloTreg_7	rec-7-2	6400	N/A	15114087	579	1040
**liverTreg_7-3_noGvHD**	liver	alloTreg_7	rec-7-3	1800	N/A	14457119	460	801
**liverTreg_6-4_noGvHD**	liver	polyTreg_6	rec-6-4	2000	N/A	15222230	450	859
**liverTreg_6-5_noGvHD**	liver	polyTreg_6	rec-6-5	5700	N/A	15483115	1089	2180
**liverTreg_6-6_noGvHD**	liver	polyTreg_6	rec-6-6	3900	N/A	15353638	914	1684
**liverTreg_7-4_noGvHD**	liver	polyTreg_7	rec-7-4	5300	N/A	14268740	729	1142
**liverTreg_7-5_noGvHD**	liver	polyTreg_7	rec-7-5	3000	N/A	14425794	322	556
**liverTreg_7-6_noGvHD**	liver	polyTreg_7	rec-7-6	1800	N/A	14968118	208	340
**spleenTreg_6-1_noGvHD**	spleen	alloTreg_6	rec-6-1	24600	8.6	15438565	5873	6622
**spleenTreg_6-2_noGvHD**	spleen	alloTreg_6	rec-6-2	14600	9.3	15339047	12998	28901
**spleenTreg_6-3_noGvHD**	spleen	alloTreg_6	rec-6-3	6600	9.1	14795306	8260	15345
**spleenTreg_7-1_noGvHD**	spleen	alloTreg_7	rec-7-1	50000	9.7	15067213	11163	14095
**spleenTreg_7-2_noGvHD**	spleen	alloTreg_7	rec-7-2	28000	N/A	14144894	4906	6743
**spleenTreg_7-3_noGvHD**	spleen	alloTreg_7	rec-7-3	9100	N/A	16106736	3195	4382
**spleenTreg_6-4_noGvHD**	spleen	polyTreg_6	rec-6-4	22000	N/A	14758724	2259	3555
**spleenTreg_6-5_noGvHD**	spleen	polyTreg_6	rec-6-5	14000	9.4	14404572	7790	12551
**spleenTreg_6-6_noGvHD**	spleen	polyTreg_6	rec-6-6	20000	8.9	14896738	10546	16387
**spleenTreg_7-4_noGvHD**	spleen	polyTreg_7	rec-7-4	50000	9.7	14695681	8907	12791
**spleenTreg_7-5_noGvHD**	spleen	polyTreg_7	rec-7-5	21000	N/A	13614052	4072	6393
**spleenTreg_7-6_noGvHD**	spleen	polyTreg_7	rec-7-6	40000	9.7	15510988	6022	8780

Supplemental Table 7
RNA- and TCR-sequencing data of re-isolated Treg – prophylaxis BMTs (GvHD) (accession IDs: RNA GSE223796, TCR GSE223797)

Name	Source	BMT Donor	Recipient	Cell Count (RNA)	RIN	Transcriptome (Unique Reads)	*Tcra* MIGs	*Tcrb* MIGs
colonTreg_2-1_GvHD	colon	alloTreg_2	rec-2-1	4200	N/A	17533986	540	611
colonTreg_2-2_GvHD	colon	alloTreg_2	rec-2-2	3580	N/A	13657886	287	333
colonTreg_2-3_GvHD	colon	alloTreg_2	rec-2-3	3100	N/A	7785484	298	367
colonTreg_3-1_GvHD	colon	alloTreg_3	rec-3-1	20000	6.6	12164994	1224	959
colonTreg_3-2_GvHD	colon	alloTreg_3	rec-3-2	13200	N/A	19823598	2211	1956
colonTreg_3-3_GvHD	colon	alloTreg_3	rec-3-3	18000	8.9	11878018	289	249
colonTreg_2-4_GvHD	colon	polyTreg_2	rec-2-4	3400	N/A	11859032	382	302
colonTreg_2-5_GvHD	colon	polyTreg_2	rec-2-5	3000	N/A	8649635	388	354
colonTreg_2-6_GvHD	colon	polyTreg_2	rec-2-6	7100	N/A	11279882	896	1195
colonTreg_3-4_GvHD	colon	polyTreg_3	rec-3-4	30400	9.2	12063227	4418	3384
colonTreg_3-5_GvHD	colon	polyTreg_3	rec-3-5	37000	9	12016899	3813	3965
colonTreg_3-6_GvHD	colon	polyTreg_3	rec-3-6	30000	8.2	12000678	3046	3298
liverTreg_2-1_GvHD	liver	alloTreg_2	rec-2-1	20900	8.7	10699097	2729	3496
liverTreg_2-2_GvHD	liver	alloTreg_2	rec-2-2	17300	6.6	11870499	2545	3083
liverTreg_2-3_GvHD	liver	alloTreg_2	rec-2-3	14400	9.5	11768793	1487	2058
liverTreg_3-1_GvHD	liver	alloTreg_3	rec-3-1	14600	6.6	12178852	1457	1677
liverTreg_3-2_GvHD	liver	alloTreg_3	rec-3-2	16000	7.5	17202929	1451	1655
liverTreg_3-3_GvHD	liver	alloTreg_3	rec-3-3	27000	9.6	14920769	2042	2232
liverTreg_2-4_GvHD	liver	polyTreg_2	rec-2-4	11400	9.4	11908583	1879	2398
liverTreg_2-5_GvHD	liver	polyTreg_2	rec-2-5	7200	N/A	43468279	1483	1653
liverTreg_2-6_GvHD	liver	polyTreg_2	rec-2-6	15500	8.1	12000202	2114	2647
liverTreg_3-4_GvHD	liver	polyTreg_3	rec-3-4	12200	8.9	N/A	726	835
liverTreg_3-5_GvHD	liver	polyTreg_3	rec-3-5	13500	7.9	11885454	1232	1665
liverTreg_3-6_GvHD	liver	polyTreg_3	rec-3-6	20600	9.3	12248656	1404	1787
spleenTreg_2-1_GvHD	spleen	alloTreg_2	rec-2-1	115700	9.6	12236022	42501	48124
spleenTreg_2-2_GvHD	spleen	alloTreg_2	rec-2-2	102000	9.4	14264559	25007	28421
spleenTreg_2-3_GvHD	spleen	alloTreg_2	rec-2-3	142000	9.3	12459864	41840	38866
spleenTreg_3-1_GvHD	spleen	alloTreg_3	rec-3-1	100000	9.5	13017042	28422	21458
spleenTreg_3-2_GvHD	spleen	alloTreg_3	rec-3-2	120000	9.4	11208441	69539	55169
spleenTreg_3-3_GvHD	spleen	alloTreg_3	rec-3-3	111000	9.6	12308467	58713	48219
spleenTreg_2-4_GvHD	spleen	polyTreg_2	rec-2-4	85700	9.7	12082745	20022	22752
spleenTreg_2-5_GvHD	spleen	polyTreg_2	rec-2-5	30000	9.7	12005090	6591	6922
spleenTreg_2-6_GvHD	spleen	polyTreg_2	rec-2-6	105000	9.6	11963245	19710	26768
spleenTreg_3-4_GvHD	spleen	polyTreg_3	rec-3-4	50000	9.6	11878880	9903	10647
spleenTreg_3-5_GvHD	spleen	polyTreg_3	rec-3-5	60000	9.7	11970871	10210	11277
spleenTreg_3-6_GvHD	spleen	polyTreg_3	rec-3-6	50000	9.3	11941935	6283	7089

Supplemental Table 8
Cell counts in colon/spleen/liver/donor clusters
(accession ID: scRNA-seq & scTCR-seq GSE223798)

CLUSTER	DONOR	COLON 1	COLON 2	COLON 3	LIVER 1	LIVER 2	LIVER 3	SPLEEN 1	SPLEEN 2	SPLEEN 3
1		2946<br>35.42%	2700<br>32.46%	2641<br>31.75%		1<br>0.01%	26<br>0.31%	2<br>0.02%		1<br>0.01%
2	1<br>0.01%	42<br>0.63%	38<br>0.57%	34<br>0.51%	1594<br>23.88%	1494<br>22.38%	1077<br>16.13%	761<br>11.4%	934<br>13.99%	700<br>10.49%
3		2071<br>33.26%	1996<br>32.06%	2112<br>33.92%	5<br>0.08%	2<br>0.03%	30<br>0.48%	3<br>0.05%	2<br>0.03%	5<br>0.08%
4	1<br>0.03%	6<br>0.16%	7<br>0.19%	7<br>0.19%	188<br>5.06%	168<br>4.52%	143<br>3.85%	994<br>26.73%	1196<br>32.17%	1008<br>27.11%
5		11<br>0.31%	19<br>0.54%	14<br>0.4%	159<br>4.51%	170<br>4.83%	91<br>2.58%	980<br>27.83%	1206<br>34.24%	872<br>24.76%
6		6<br>0.17%	14<br>0.4%	9<br>0.26%	585<br>16.82%	582<br>16.73%	407<br>11.7%	609<br>17.51%	728<br>20.93%	538<br>15.47%
7	2816<br>99.89%			1<br>0.04%	1<br>0.04%					1<br>0.04%
8		5<br>0.59%		1<br>0.12%	50<br>5.91%	52<br>6.15%	9<br>1.06%	312<br>36.88%	272<br>32.15%	145<br>17.14%
9		37<br>4.82%	23<br>2.99%	37<br>4.82%	120<br>15.62%	104<br>13.54%	71<br>9.24%	102<br>13.28%	185<br>24.09%	89<br>11.59%
10		3<br>0.72%	5<br>1.2%	4<br>0.96%	6<br>1.44%	7<br>1.67%	3<br>0.72%	105<br>25.12%	76<br>18.18%	209<br>50%
11		126<br>39.5%	101<br>31.66%	88<br>27.59%			3<br>0.94%	1<br>0.31%		
12	248<br>99.6%							1<br>0.4%		
13								9<br>8.82%	51<br>50%	42<br>41.18%

Supplemental Table 9
Cell counts in colon clusters
(accession ID: scRNA-seq & scTCR-seq GSE223798)

CLUSTER	COLON 1	COLON 2	COLON 3
1	1277<br>35%	1188<br>32.56%	1184<br>32.45%
2	974<br>34.39%	948<br>33.47%	910<br>32.13%
3	808<br>34.11%	829<br>34.99%	732<br>30.9%
4	746<br>31.96%	686<br>29.39%	902<br>38.65%
5	778<br>37.17%	711<br>33.97%	604<br>28.86%
6	271<br>41.89%	202<br>31.22%	174<br>26.89%
7	154<br>30.56%	157<br>31.15%	193<br>38.29%
8	135<br>38.24%	76<br>21.53%	142<br>40.23%

Supplemental Table 10
scRNA-seq QC metrics
(accession ID: GSE223798)

Sample	Number of Reads	Estimated Number of Cells	Mean Reads per Cell	Median Genes per Cell	Valid Barcodes	Reads Mapped to Genome	Fraction Reads in Cells	Total Genes Detected	Median UMI Counts per Cell	Cells per cluster	Cells with TCR
Donor allo	376523583	5560	67720	2332	87.5 %	91.5 %	82.9 %	16567	9369	3066	2037
Colon recipient 1	442390212	7866	56240	1735	86.0 %	91.3 %	81.7 %	17297	4491	5253	3530
Liver recipient 1	345854859	5336	64815	1672	88.6 %	90.9 %	66.1 %	16328	3992	2708	1889
Spleen recipient 1	463924730	6676	69491	1826	86.2 %	91.2 %	76.6 %	16564	4740	3879	2795
Colon recipient 2	455731582	6832	66705	1671	88.2 %	90.5 %	86.4 %	17047	4353	4903	3345
Liver recipient 2	281835717	4633	60832	1659	89.5 %	91.6 %	72.3 %	16124	4064	2580	1841
Spleen recipient 2	406491956	7703	52770	1649	89.2 %	91.0 %	76.9 %	16683	4256	4650	3249
Colon recipient 3	437143148	6593	66304	1739	86.4 %	92.3 %	89.6 %	16721	4574	4948	3317
Liver recipient 3	260585726	3690	70619	1625	87.5 %	91.5 %	67.7 %	15809	3963	1860	1329
Spleen recipient 3	385355848	6265	61509	1813	85.3 %	91.8 %	78.4 %	17095	4755	3610	2557

Supplemental Table 11
scTCR-seq QC metrics
(accession ID: GSE223798)

Sample Name	Number of Read Pairs	Estimated Number of Cells	Mean Read Pairs per Cell	Number of Cells With Productive V-J Spanning Pair	Valid Barcodes	Reads Mapped to Any V(D)J Gene	Reads Mapped to Tcra	Reads Mapped to Tcrb	Fraction Reads in Cells
Donor allo*	132123300	19314	6840	18318	95.5 %	89.0 %	27.2 %	61.8 %	45.6 %
Donor allo	34278811	5124	6689	4501	96.8 %	93.4 %	23.1 %	70.2 %	65.5 %
Colon recipient 1	40069704	6876	5827	5915	97.1 %	93.5 %	36.0 %	57.4 %	74.5 %
Liver recipient 1	36121241	5294	6823	4376	97.5 %	94.8 %	25.5 %	69.2 %	58.9 %
Spleen recipient 1	54058522	6287	8598	5595	97.2 %	94.5 %	30.4 %	64.1 %	68.5 %
Colon recipient 2	55842398	5906	9455	5133	97.5 %	94.0 %	35.8 %	58.2 %	79.0 %
Liver recipient 2	33139445	4330	7653	3724	97.5 %	94.4 %	27.3 %	67.0 %	62.6 %
Spleen recipient 2	38079303	6918	5504	6039	97.5 %	94.8 %	31.1 %	63.7 %	67.5 %
Colon recipient 3	40687781	5734	7095	5039	97.2 %	94.0 %	35.4 %	58.6 %	76.1 %
Liver recipient 3	25524413	3491	7311	2921	97.4 %	94.6 %	26.8 %	67.7 %	58.2 %
Spleen recipient 3	44480090	5528	8046	4974	97.0 %	93.6 %	30.3 %	63.2 %	69.7 %

Supplemental References

1. Miragaia RJ*, et al.* Single-Cell Transcriptomics of Regulatory T Cells Reveals Trajectories of Tissue Adaptation. *Immunity* **50**, 493-504.e497 (2019).

2. Aubert N, Salomon BL, Marodon G. Characterization of a regulatory T cells molecular meta-signature identifies the pro-enkephalin gene as a novel marker in mice. *bioRxiv*, 638072 (2020).

3. Chen PP*, et al.* Alloantigen-specific type 1 regulatory T cells suppress through CTLA-4 and PD-1 pathways and persist long-term in patients. *Sci Transl Med* **13**, eabf5264 (2021).

4. Shalev I*, et al.* Targeted deletion of fgl2 leads to impaired regulatory T cell activity and development of autoimmune glomerulonephritis. *J Immunol* **180**, 249-260 (2008).

5. Shevach EM. Mechanisms of foxp3+ T regulatory cell-mediated suppression. *Immunity* **30**, 636-645 (2009).

6. Schmidt A, Oberle N, Krammer PH. Molecular mechanisms of treg-mediated T cell suppression. *Front Immunol* **3**, 51 (2012).

7. van der Veeken J*, et al.* Genetic tracing reveals transcription factor Foxp3-dependent and Foxp3-independent functionality of peripherally induced Treg cells. *Immunity* **55**, 1173-1184 e1177 (2022).

8. Liberzon A, Birger C, Thorvaldsdottir H, Ghandi M, Mesirov JP, Tamayo P. The Molecular Signatures Database (MSigDB) hallmark gene set collection. *Cell Syst* **1**, 417-425 (2015).
