# Supplementary material for: Donor regulatory T cells rapidly adapt to recipient tissues to control murine acute graft-versus-host disease
Source: Nat Commun. 2024 Apr 15;15:3224. doi: 10.1038/s41467-024-47575-z (PMC11018811; doi:10.1038/s41467-024-47575-z)
Supplement: Supplementary file 3 — Reporting Summary [file 41467_2024_47575_MOESM3_ESM.pdf]

nature portfolio

Corresponding author(s): Petra Hoffmann, Matthias Edinger, Michael Rehli

Last updated by author(s): Mar 6, 2024

Reporting Summary

Nature Portfolio wishes to improve the reproducibility of the work that we publish. This form provides structure for consistency and transparency in reporting. For further information on Nature Portfolio policies, see our Editorial Policies and the Editorial Policy Checklist.

Statistics

For all statistical analyses, confirm that the following items are present in the figure legend, table legend, main text, or Methods section.

n/a	Confirmed	
☐	☒	The exact sample size (*n*) for each experimental group/condition, given as a discrete number and unit of measurement
☐	☒	A statement on whether measurements were taken from distinct samples or whether the same sample was measured repeatedly
☐	☒	The statistical test(s) used AND whether they are one- or two-sided<br>*Only common tests should be described solely by name; describe more complex techniques in the Methods section.*
☒	☐	A description of all covariates tested
☐	☒	A description of any assumptions or corrections, such as tests of normality and adjustment for multiple comparisons
☐	☒	A full description of the statistical parameters including central tendency (e.g. means) or other basic estimates (e.g. regression coefficient) AND variation (e.g. standard deviation) or associated estimates of uncertainty (e.g. confidence intervals)
☐	☒	For null hypothesis testing, the test statistic (e.g. *F*, *t*, *r*) with confidence intervals, effect sizes, degrees of freedom and *P* value noted<br>*Give P values as exact values whenever suitable.*
☒	☐	For Bayesian analysis, information on the choice of priors and Markov chain Monte Carlo settings
☐	☒	For hierarchical and complex designs, identification of the appropriate level for tests and full reporting of outcomes
☒	☐	Estimates of effect sizes (e.g. Cohen's *d*, Pearson's *r*), indicating how they were calculated

Our web collection on statistics for biologists contains articles on many of the points above.

Software and code

Policy information about availability of computer code

Data collection	Flow cytometry: FACSDiva™ (BD Biosciences) v8.0.1 (FACSAria IIu), v8.0.3 FACSAria Fusion), v9.1 (FACSymphony)
Data analysis	FlowJo® (v10.7.1 - v10.8.1; Treestar Inc); Adobe Illustrator (v25.2.1); bcl2fastq Conversion Software (v1.8.4); STAR v2.5.3a; Graphia (v.3.0); R (v4.0.3-4.1.0); PEAR (v0.9.11); MIGEC (v1.2.9); MIXCR (v3.0.13); cellranger mkfastq (4.0.0); cellranger count (4.0.0); kallisto-bustools (kb-python 0.27.2); scvelo (0.2.4); cellranger (version 5.0.0);<br>R packages: survival (v3.2-13), survminer (v0.4.9), edgeR (v3.34.0), ggplot2 (v3.3.3-3.3.5), ggrepel (v0.9.1), umap (v0.2.7.0), limma (v3.48.1), pheatmap (v1.0.12), gplots (v3.1.1), immunarch (v.0.6.6), circlize (0.4.12), Seurat (v4.0.0), Doubletfinder (2.0.3)

For manuscripts utilizing custom algorithms or software that are central to the research but not yet described in published literature, software must be made available to editors and reviewers. We strongly encourage code deposition in a community repository (e.g. GitHub). See the Nature Portfolio guidelines for submitting code & software for further information.

Data

Policy information about availability of data

All manuscripts must include a data availability statement. This statement should provide the following information, where applicable:

- Accession codes, unique identifiers, or web links for publicly available datasets
- A description of any restrictions on data availability
- For clinical datasets or third party data, please ensure that the statement adheres to our policy

Raw and processed sequencing data are deposited with the Gene Expression Omnibus (GEO) data repository (GSE223800, https://www.ncbi.nlm.nih.gov/geo/query/acc.cgi?acc=GSE223800). The reference mouse genome assembly (Release M16, GRCm38.p5) was retrieved from Gencode (https://www.gencodegenes.org). Source data are provided with this paper.

Research involving human participants, their data, or biological material

Policy information about studies with human participants or human data. See also policy information about sex, gender (identity/presentation), and sexual orientation and race, ethnicity and racism.

Reporting on sex and gender	n/a
Reporting on race, ethnicity, or other socially relevant groupings	n/a
Population characteristics	n/a
Recruitment	n/a
Ethics oversight	n/a

Note that full information on the approval of the study protocol must also be provided in the manuscript.

Field-specific reporting

Please select the one below that is the best fit for your research. If you are not sure, read the appropriate sections before making your selection.

- [x] Life sciences
- [ ] Behavioural & social sciences
- [ ] Ecological, evolutionary & environmental sciences

For a reference copy of the document with all sections, see nature.com/documents/nr-reporting-summary-flat.pdf

Life sciences study design

All studies must disclose on these points even when the disclosure is negative.

Sample size	Statistical methods were not applied to predetermine sample size. Similar studies in the field, previous experiences, balancing statistical robustness and animal welfare were used to determine sample sizes . For bulk sequencing experiments we performed two sets of transplantations into three animals each. The single cell sequencing experiment included one set of transplantations into three animals. Cells from each animal were harvested separately, hence, we used between three to six biological replicates of the data that we collected, as indicated. Since replicates generally aligned well, we consider sample sizes sufficient. Sample sizes for FACS were generally in the same range (3 independent experiments/transplantations; cells pooled from n=3 animals per group and experiment). However, the number of markers that could be determined per experiment varied with the number of re-isolated cells. Hence in some cases we also report sample sizes <3.
Data exclusions	All data are provided, and no data points were excluded. Sample sizes are stated in the respective figure legends
Replication	Experimental findings were reliably reproduced. The number of independent experiments and biological replicates are stated in the respective figure legends.
Randomization	Animals were randomly allocated into the BMT groups (no GvHD/GvHD; allo or polyTreg)
Blinding	Except for the clinical GvHD scoring, only objective data were collected and thus blinding was not applicable. Personnel involved in clinical GvHD scoring was not involved in study planning and thus unbiased.

Reporting for specific materials, systems and methods

We require information from authors about some types of materials, experimental systems and methods used in many studies. Here, indicate whether each material, system or method listed is relevant to your study. If you are not sure if a list item applies to your research, read the appropriate section before selecting a response.

Materials & experimental systems

n/a	Involved in the study
	[x] Antibodies
[x]	Eukaryotic cell lines
[x]	Palaeontology and archaeology
	[x] Animals and other organisms
[x]	Clinical data
[x]	Dual use research of concern
[x]	Plants

Methods

n/a	Involved in the study
[x]	ChIP-seq
	[x] Flow cytometry
[x]	MRI-based neuroimaging

Antibodies

Antibodies used

ANTIGEN , CLONE ,DILUTION/AMOUNT , FLUOROCHROME(S) (SOURCE, CATALOG NUMBER)
Biotin , REA746 ,2 µl per test , PerCP (Miltenyi Biotec, 130-110-960)
CD3 (NA/LE) ,145-2C11 ,Final conc. per well: 0.4 µg/ml , - (BD Biosciences, 553057)
CD4, RM4-5 , 1:200 , PB (BD Biosciences, 558107)
CD4, RM4-5 , 1:100 , BV510 (BD Biosciences, 563106)
CD4, RM4-5 , 1:133.3 , BV605 (BD Biosciences, 563151)
CD8a, 53-6.7 , 1:100 , BUV805 (BD Biosciences, 612898)
CD8a, 53-6.7 , 1:100 , PerCP-Cy5-5 (BD Biosciences, 551162)
CD11b,M1/70 , 1:1000 , PE (eBioscience, 12-0112-82)
CD11b,M1/70 , 1:200 , PE-Cy7 (BD Biosciences,552850)
CD11c, N418 , 1:100 , PerCP (BioLegend, 117326)
CD16/32,93 , 1:50 , Purified (BioLegend,101302)
CD19, 1D3 , 1:133.3 , BV650 (BD Biosciences,563235)
CD19, 1D3 , 1:133.3 , FITC (BD Biosciences, 553785)
CD25, PC61 , 1:100 , PE (BD Biosciences, 553866)
CD25, PC61.5 , 1:200 , PE-Cy7 (eBioscience, 25-0251-82)
CD45, 30F-11 , 1:200 , BV510 (BD Biosciences, 563891)
CD45.1, A20 , 1:100 , BUV395 (BD Biosciences, 565212)
CD45.1, A20 , 1:200 , Biotin (BioLegend, 110704)
CD45.2,104 , 1:200 , PB (BioLegend, 109820)
CD45.2,104 , 1:100 , BV786 (BD Biosciences, 563686)
CD62L, MEL-14 , 1:133.3 , APC (BD Biosciences, 553152)
CD103,2E7 , 1:80 , APC (eBioscience, 17-1031-82)
CD199/CCR9, EBIOCW-1.2 , 1:200 , PE-CY7 (eBioscience, 17-1991-82)
FOXP3, FJK-16s , 1:100 , FITC (eBioscience, 11-5773-82)
GR-1, RB6-8C5 , 1:200 , APC (BD Biosciences, 553129)
H-2Kb, AF6-88.5 , 1:100 , Biotin (BioLegend, 116504)
H-2Kb, AF6-88.5 , 1:100 , PE (BioLegend, 116508)
HELIOS, 22F6 , 1:20 , PE (BioLegend, 137216)
KI-67, SolA15 , 1:2000 , eFluor660 (eBioscience, 50-5698-82)
KLRG-1, 2F1/KLRG1 , 1:100 , BV605 (BioLegend, 138419)
LY6C, HK1.4 , 1:100 , PB (BioLegend, 128014)
LPAM-1, DATK32 , 1:80 , PE (BD Biosciences, 553811)
NKP46, 29A1.4 , 1:25 , BV510 (BD Biosciences, 563455)
TCRβ, H57-597 , 1:200 , PE (BD Biosciences, 553172)
TCRβ, H57-597 , 1:50 , BUV737 (BD Biosciences, 612821)
TCRβ, REA318 , 1:20 , APC (Miltenyi Biotec, 130-104-810)
TER-119, TER-119 , 1:100 , APC-eF780 (eBioscience, 47-5921-82)

Validation

All antibodies used in this study are monoclonal and commercially availabe. Validation of species and antigen specificity is provided by supplier:

BIOTIN, REA746, PerCP (Miltenyi Biotec): https://www.miltenyibiotec.com/DE-en/products/biotin-antibody-reafinity-rea746.html
CD3, (NA/LE), 145-2C11, - (BD Biosciences): https://www.bdbiosciences.com/en-pt/products/reagents/flow-cytometry-reagents/research-reagents/single-color-antibodies-ruo/purified-na-le-hamster-anti-mouse-cd3e.553057
CD4, RM4-5, PB, BV510, BV605 (BD Biosciences): https://www.bdbiosciences.com/en-in/products/reagents/flow-cytometry-reagents/research-reagents/single-color-antibodies-ruo/bv605-rat-anti-mouse-cd4.563151
CD8a, 53-6.7, BUV805, PerCP-Cy5-5 (BD Biosciences): https://www.bdbiosciences.com/en-us/products/reagents/flow-cytometry-reagents/research-reagents/single-color-antibodies-ruo/percp-cy-5-5-rat-anti-mouse-cd8a.561109
CD11b, M1/70 PE (eBioscience), PE-Cy7 (BD Biosciences): https://www.thermofisher.com/antibody/product/CD11b-Antibody-clone-M1-70-Monoclonal/12-0112-82; https://www.bdbiosciences.com/en-us/products/reagents/flow-cytometry-reagents/research-reagents/single-color-antibodies-ruo/pe-cy-7-rat-anti-cd11b.561098
CD11c, N418, PerCP, (BioLegend): https://www.biolegend.com/de-de/cell-health/percp-cyanine5-5-anti-mouse-cd11c-antibody-4258

CD16/32, 93, Purified (BioLegend): https://www.biolegend.com/ja-jp/products/purified-anti-mouse-cd16-32-antibody-190?GroupID=BLG9237
CD19, 1D3, BV650, FITC (BD Biosciences): https://www.bdbiosciences.com/en-us/products/reagents/flow-cytometry-reagents/research-reagents/single-color-antibodies-ruo/fitc-rat-anti-mouse-cd19.553785
CD25, PC61, PE (BD Biosciences): https://www.bdbiosciences.com/en-br/products/reagents/flow-cytometry-reagents/research-reagents/single-color-antibodies-ruo/pe-rat-anti-mouse-cd25.553866
CD25, PC61.5, PE-Cy7, (eBioscience): https://www.thermofisher.com/antibody/product/CD25-Antibody-clone-PC61-5-Monoclonal/25-0251-82
CD45, 30F-11, BV510 (BD Biosciences): https://www.bdbiosciences.com/en-br/products/reagents/flow-cytometry-reagents/research-reagents/single-color-antibodies-ruo/bv510-rat-anti-mouse-cd45.563891
CD45.1, A20, BUV395 (BD Biosciences), Biotin (BioLegend): https://www.bdbiosciences.com/en-us/products/reagents/flow-cytometry-reagents/research-reagents/single-color-antibodies-ruo/buv395-mouse-anti-mouse-cd45-1.565212; https://www.biolegend.com/en-ie/products/biotin-anti-mouse-cd45-1-antibody-197
CD45.2, 104, PB (BioLegend), BV786 (BD Biosciences): https://www.biolegend.com/de-at/products/pacific-blue-anti-mouse-cd45-2-antibody-3108; https://www.bdbiosciences.com/en-us/products/reagents/flow-cytometry-reagents/research-reagents/single-color-antibodies-ruo/bv786-mouse-anti-mouse-cd45-2.563686
CD62L, MEL-14, APC (BD Biosciences): https://www.bdbiosciences.com/en-lu/products/reagents/flow-cytometry-reagents/research-reagents/single-color-antibodies-ruo/apc-rat-anti-mouse-cd62l.553152
CD103, 2E7, APC (eBioscience): https://www.thermofisher.com/antibody/product/17-1031-82.html
CD199/CCR9, EBIOCW-1.2, PE-CY7 (eBioscience): https://www.thermofisher.com/antibody/product/CD199-CCR9-Antibody-clone-eBioCW-1-2-CW-1-2-Monoclonal/25-1991-82
FOXP3, FJK-16s, FITC (eBioscience): https://www.thermofisher.com/antibody/product/11-5773-82.html
GR-1, RB6-8C5, APC (BD Biosciences): https://www.bdbiosciences.com/en-eu/products/reagents/flow-cytometry-reagents/research-reagents/single-color-antibodies-ruo/apc-rat-anti-mouse-ly-6g-and-ly-6c.553129
H-2Kb, AF6-88.5, Biotin, PE (BioLegend): https://www.biolegend.com/fr-lu/products/biotin-anti-mouse-h-2kb-antibody-1747?GroupID=BLG2539
HELIOS, 22F6, PE (BioLegend): https://www.biolegend.com/de-de/products/pe-anti-mouse-human-helios-antibody-6481?Clone=22F6
KI-67, SolA15, eFluor660 (eBioscience): https://www.thermofisher.com/antibody/product/Ki-67-Antibody-clone-SolA15-Monoclonal/50-5698-82
KLRG-1, 2F1/KLRG1, BV605 (BioLegend): https://www.biolegend.com/de-at/products/brilliant-violet-605-anti-mouse-human-klrg1-mafa-antibody-9644?GroupID=BLG8908
LY6C, HK1.4, PB (BioLegend): https://www.biolegend.com/fr-fr/products/pacific-blue-anti-mouse-ly-6c-antibody-6024?GroupID=BLG7242
LPAM-1, DATK32, PE (BD Biosciences): https://www.bdbiosciences.com/en-nz/products/reagents/flow-cytometry-reagents/research-reagents/single-color-antibodies-ruo/pe-rat-anti-mouse-lpam-1.553811
NKP46, 29A1.4, BV510 (BD Biosciences): https://www.bdbiosciences.com/en-us/products/reagents/flow-cytometry-reagents/research-reagents/single-color-antibodies-ruo/bv510-rat-anti-mouse-cd335-nkp46.563455
TCRβ, H57-597, PE, BUV737 (BD Biosciences): https://www.bdbiosciences.com/en-us/products/reagents/flow-cytometry-reagents/research-reagents/single-color-antibodies/buv737-hamster-anti-mouse-tcr-chain.612821
TCRβ, REA318, APC (Miltenyi Biotec): https://www.miltenyibiotec.com/DE-en/products/tcrb-antibody-anti-mouse-reafinity-rea318.html#conjugate=apc:size=30-ug-in-200-ul
TER-119, TER-119, APC-eF780 (eBioscience): https://www.thermofisher.com/antibody/product/TER-119-Antibody-clone-TER-119-Monoclonal/47-5921-82

Animals and other research organisms

Policy information about studies involving animals; ARRIVE guidelines recommended for reporting animal research, and Sex and Gender in Research

Laboratory animals	species: Mus musculus; strains: BALB/cAnNCrl (H-2d), C57BL/6NCrl (H-2b) , B6.SJL-Ptprca Pepcb/BoyJ (CD45.1), FoxP3EGFP (B. Malissen); age: 8–12 wks (BMT donors), 11–16 wks (BMT recipients).
Wild animals	No wild animals were used in the study.
Reporting on sex	In line with similar studies in the field, only female animals were used as donors and recipients in the study. Female recipients were used for experimental reasons (randomization before the start of the experiment), female donors were used as male into female transplantation would have introduced an additional variable. There are no indications that the main findings of this study apply only to female donor/recipient pairs.
Field-collected samples	No field collected samples were used in the study.
Ethics oversight	Animal studies were approved by the Committee on Ethics of Animal Experiments at the Bavarian Government (Ref-No: 55.2-2532-2-430).

Note that full information on the approval of the study protocol must also be provided in the manuscript.

Plants

Seed stocks	n/a
Novel plant genotypes	n/a
Authentication	n/a

Flow Cytometry

Plots

Confirm that:

- [x] The axis labels state the marker and fluorochrome used (e.g. CD4-FITC).
- [x] The axis scales are clearly visible. Include numbers along axes only for bottom left plot of group (a 'group' is an analysis of identical markers).
- [ ] All plots are contour plots with outliers or pseudocolor plots.
- [x] A numerical value for number of cells or percentage (with statistics) is provided.

Methodology

Sample preparation	Single cell suspensions from BM, spleen and mesenteric lymph nodes were prepared and erythrocytes were lysed. Leukocyte isolation from liver: organ was flushed with PBS through the portal vein and the gallbladder removed before excision, dissected into small pieces, fragments suspended in RPMI /5% FCS, strained and washed before Percoll centrifugation (40%/80%). Leukocytes were resuspended in RPMI/5% FCS, erythrocytes lysed, cells washed and kept on ice. For intestinal leukocyte isolation, large intestine was excised, cut into 0.5 cm pieces, incubated twice for 20 min at 37 °C in HBSS/5mM EDTA/1mM DTT followed by vigorous shaking to isolate intraepithelial leukocytes (IEL). For lamina propria leukocytes (LPL), the fragments were transferred to HBSS w/o phenol red containing calcium and magnesium with 5% FCS together with enzymes of the Lamina propria dissociation kit (Miltenyi Biotec) and incubated for 30 min at 37°C. The fragments were further dissociated using the GentleMACS system (protocol: m_intestine_01; Miltenyi Biotec), strained, washed, and pooled with IEL before Percoll centrifugation (30%).
Instrument	Analysis: FACSymphony™ A5 SORP (Becton Dickinson); Sort:FACSAria™ IIu or a FACSAria™ Fusion (Becton Dickinson)
Software	Data collection: FACSDiva™ (BD Biosciences) v8.0.1 (FACSAria™ IIu), v8.0.3 (FACSAria™ Fusion), v9.1 (FACSymphony™)<br>Data analysis: FlowJo® v10.7.1/v10.8.0 (Treestar Inc.,Ashland, OR).
Cell population abundance	Sort purity was determined by re-analysis of sorted material on the cell sorter. Sample purity of sorted CD4+CD8-CD25 +CD62L+ cells (starting population for Treg cultures) was above 95%. Treg for bulk RNASeq were sorted directly into lysis buffer. Purity of random samples was above 95%.
Gating strategy	Starting cell population was gated as follows: a) FSc/SSc according to leucocyte characteristics, b) live gate (exclusion of DAPI+ or Live/Dead+ cells), c) gating on single cells (FSc-A vs FSc-H). Further gating steps are shown in detail in the supplemental information. Thresholds for positive staining are defined by intra or inter sample marker-negative reference populations.

- [x] Tick this box to confirm that a figure exemplifying the gating strategy is provided in the Supplementary Information.
